# Thin-Wall Machining of Light Alloys: A Review of Models and Industrial Approaches

**DOI:** 10.3390/ma12122012

**Published:** 2019-06-23

**Authors:** Irene Del Sol, Asuncion Rivero, Luis Norberto López de Lacalle, Antonio Juan Gamez

**Affiliations:** 1 Department of Mechanical Engineering and Industrial Design, Faculty of Engineering, Universidad de Cádiz, 11519 Puerto Real, Spain; antoniojuan.gamez@uca.es; 2 Tecnalia Research & Innovation. Scientific and Technological Park of Guipuzkoa, 20009 Donostia-San Sebastian, Spain; asun.rivero@tecnalia.com; 3 Department of Mechanical Engineering, University of the Basque Country, 48013 Bilbao, Spain; norberto.lzlacalle@ehu.eus

**Keywords:** thin-wall machining, chatter, vibration, deflection, damping, prediction, workholding, fixture, dynamic, stability

## Abstract

Thin-wall parts are common in the aeronautical sector. However, their machining presents serious challenges such as vibrations and part deflections. To deal with these challenges, different approaches have been followed in recent years. This work presents the state of the art of thin-wall light-alloy machining, analyzing the problems related to each type of thin-wall parts, exposing the causes of both instability and deformation through analytical models, summarizing the computational techniques used, and presenting the solutions proposed by different authors from an industrial point of view. Finally, some further research lines are proposed.

## 1. Introduction

A wide range of aeronautical parts such as stringers, ribs, frames, spars, hubs, blisks, turbine blades, shells, bulkheads or skin panels can be classified as thin-wall structures [1]. They are designed to avoid mechanical assembly using bolts or rivets and to keep a uniform behavior all over the part. Thin-wall structures are usually manufactured out of advanced materials widely used in the aeronautical sector such as aluminum and titanium alloys, although some high-performance materials such as Inconel or specific types of stainless steel can also be used. Their good corrosion resistance and strength properties allow reducing the quantity of material needed, obtaining slim parts with a good weight–resistance ratio [2]. Therefore, thin-wall parts are manufactured by removing large quantities of material from the original block through a machining process, typically achieving a 30:1 buy-to-fly ratio [1]. The in-process parts are characterized by their low stiffness and constant change of mechanical properties. Their thickness is at least six times lower than the two other relevant directions, thus being flexible and easy to bend.

This fact produces dynamic and static problems during the machining operation. On the one hand, dynamic instabilities become frequent, and self-excited vibrations or chatter are more likely to appear, increasing the roughness of the final part, the tool wear and the wear of the different machine components [3,4]. Forced vibrations provoked by the dynamic of the machining operation also affect the final part, therefore reducing its quality. On the other hand, from a static point of view, both clamping and cutting forces produce elastic deformation that can affect the final dimension and the roughness of the part [5,6]. Induced residual stressed may also modify the final geometry of the part during the process [7]. Additionally, preforms or skin panels have to be accurately positioned on the machining center to ensure the tight tolerances [8].

**Figure 1:**
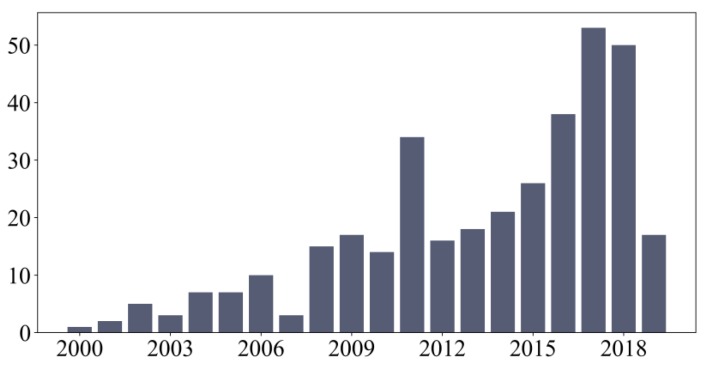
The number papers published on Web of Science related to thin-wall machining.

If these problems cannot be controlled, companies are forced to include reprocessing steps or discard useless parts, increasing the production cost. Being difficult to manufacture, thin-wall machining costs are justified just by weight reduction and fuel efficiency. For this reason, different organizations such as the European H2020 [9] have funded research projects on the improvement of thin-wall machining. Globally, this research line has been increasing, as can be appreciated by the number of papers focused on this topic published in international scientific databases (Figure 1).

To ensure thin-wall quality, several authors have proposed analytical or computational dynamic models to predict the behavior of the part and select the best parameters to reduce chatter [10] or to damp the system [2]. Others have studied the deflection of the part due to the force interaction [11] or to the induced residual stress [12].

Alternatively, industry has developed special clamping or monitoring systems combined with adaptive control, such that quality is measured on-line and statistical parameters optimizations are used to monitor periodic changes [13].

The purpose of this work is to review the state of the art of thin-wall machining. Initially, thin-wall parts are identified, classified and associated to the critical problems previously introduced. Then, to understand the dynamic and static behavior of these machining operation, the most common analytics models are exposed. The proposed solutions, focused on models or industrial approaches, are explained in different sections and the reference distribution is summarized in Table 1. Finally, conclusions are drawn and possible future research lines are proposed.

## 2. Type of Parts and Associated Problems

### 2.1. Thin-Wall Parts: Characteristics and Types

Thin-wall parts can include different types of parts, being their main characteristic their lack of stiffness and final slim factor, which is defined as their height divided by their thickness. Regarding the machining process and their characteristics, parts can be classified into two groups: monolithic blocks and skin panels.

**Figure 2:**
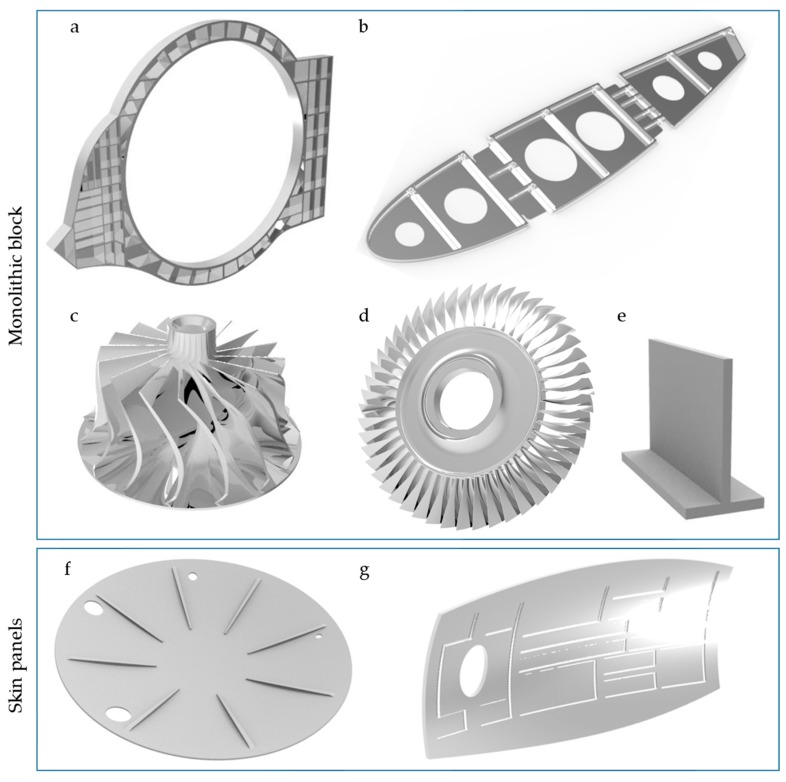
Examples of thin-wall parts: (**a**) frame; (**b**) rib; (**c**) impeller; (**d**) blisk; (**e**) sample parts; (**f**) bulkhead; and (**g**) fuselage skin.

Parts composing the first group have a geometry machined from monolithic blocks (Figure 2a–e). The part is obtained by removing a 90–95% of the initial material volume of the block by machining operations [141,142]. Stringers, ribs or frames are machined to obtain different pocket shapes, keeping their structural strength and reducing their weight. They are usually manufactured using three-axis machining, when the non-rigidity machining problems appear on the last steps for both, thin-floors and thin-walls. Impellers, blisks and blades are also included in this group, but their complex shapes require a constant change of the tool angle, changing the way the cutting forces are applied. Moreover, the cantilever produces a high deflection of the parts, making the control of the real depth of cut more difficult. Researchers use a sample part, represented in Figure 2e, as a simplification of the real cases to test and verify the machining parameters or to develop dynamic models reducing the computational time [143,144]. The behavior of the last roughing steps and end milling operation is then extrapolated to more complex shapes.

The other group, commonly known as skin panels, is mainly composed of shells, wings, fuselage parts (Figure 2g), bulkheads (Figure 2f), doors, satellite parts and frames, presenting a slim factor higher than ten [145]. Their buy-to-fly ratio is generally lower than those of monolithic parts. They are machined to pocket a large area of their surface, reducing the weight of the part. These pockets were traditionally machined using chemical milling, a highly pollutant process that does not induce residual stress and simplifies the clamping system [146]. However, since 2007, and mainly due to environmental reasons, special milling CNC centers have been designed and used for this purpose [147]. They are built with two symmetrical heads, hence the operation is named mirror milling. The machining head works perpendicular to the surface and a second head follows the machining head to ensure the support of the part and to reduce the deflection.

### 2.2. Dynamic and Static Problems

Generally, the main problem of thin-wall machining is the vibrations associated to their low rigidity. Depending on their cause, vibrations are considered self-induced (chatter) or forced.

Chatter takes place when the natural frequency response (FRF) of the system is excited due to the machining operation [14]. These instabilities are usually related to the tool vibrations produced during the machining but the most important one is the FRF of the part [17,25,27], which is constantly changing due to geometry variations. This cyclical behavior changes the FRF of the system and generates an unstable machining process [11,28]. Forced vibration or amplification takes place when the stiffness of the part is not enough to maintain a constant chip thickness. The workpiece and the tool deflect down to the edge action, producing a vibration at the same frequency as the spindle speed or multiples of it [32]. Both cases modify the contact between part and tool edge, changing the chip width, which affects the real cutting forces. Instabilities usually produce marks on the part that increase the roughness of the final product, affecting its final surface quality [45,60].

The other main problem associated to the low stiffness of the part is the dimensional error produced due to the deflection of the part, a static issue not considered on the machining of rigid parts. Machining of rigid parts usually deals with the flexibility of the cutter system [74], although it is not usually taken into account in traditional milling models. However, it is common to have an influence of the part and the cutting tool flexibility in thin-wall machining.

Static deflection can appear due to the interaction of the cutting forces [63]. In this case, the deformation usually depends on the cutting strategy (up-milling or down-milling) and the cutting parameters, which define the cutting forces and, therefore, the deformation of the system [64,66,67,68,148]. Currently, high speed milling reduces cutting forces [2,10] and induces less residual stresses [7], but sometimes this technology cannot remove the deflection completely. This fact is aggravated in mirror milling due to the real part geometry variations [75,76]. Skins come from double curve processes and their position on the clamping system do not usually match the designed one, producing overcutting areas. Additionally, those parts are larger than the used in monolithic blocks, so the workholdings and fixtures do not usually ensure the tolerances of machining for the final parts and increase the difficulty of the whole machining process.

**Figure 3:**
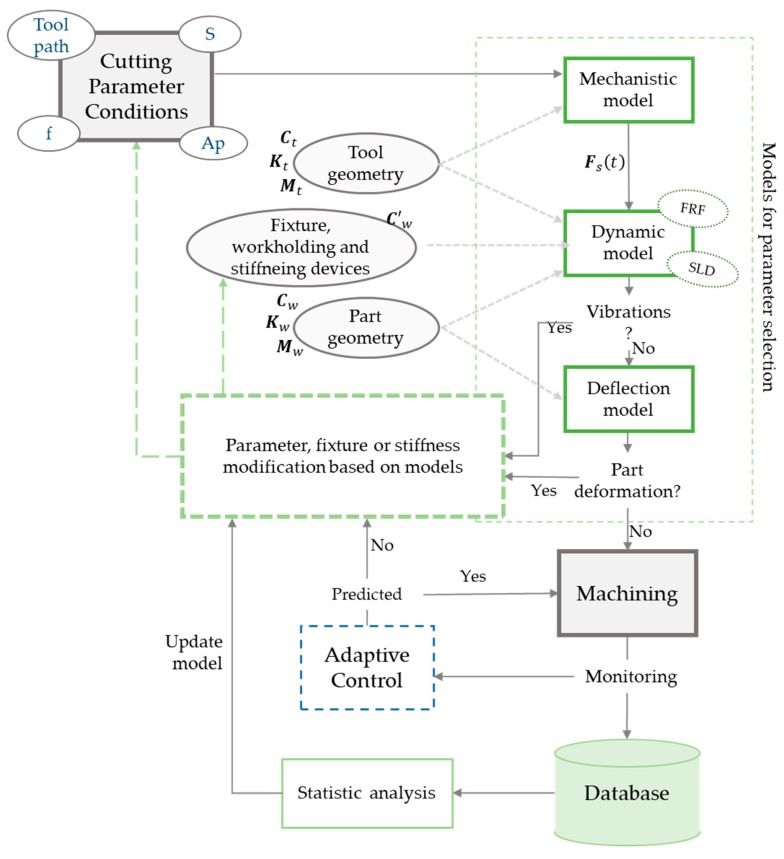
Scheme of the thin-wall machining process work flow.

The different approaches found to predict and solve both issues are summarized in Figure 3, showing a complete work flow for thin-wall machining process. It covers the sequences used in physical and statistical models, commonly applied to improve the machining performance. For design and pre-industrialization stages, computational models and virtual twins allow selecting parameters or toolpath to reduce chatter and part deformation as well as to validate the design of specific work holding and stiffening devices. In industry, different workholding, stiffening devices or adaptive control solutions based on statistics and machine learning models, have been developed following the outlines of industry 4.0.

## 3. Analytic Models

Dynamic response and machining performance of thin-wall parts were widely studied in the 1990s by Budak et al. [22,23]. Most of the research studies focused on the design of new methods to predict the behavior of the system are based on the FRF [57] and the deformations produced by the cutting forces [67]. Their final objective is the selection of the tool geometry and cutting parameters such that chip thickness is constant and vibrations are reduced. For this purpose, and to understand the physical fundamentals of thin-wall machining, analytic models of the forces, dynamics and deflection are explained in the following sections.

### 3.1. Cutting Force Prediction

**Figure 4:**
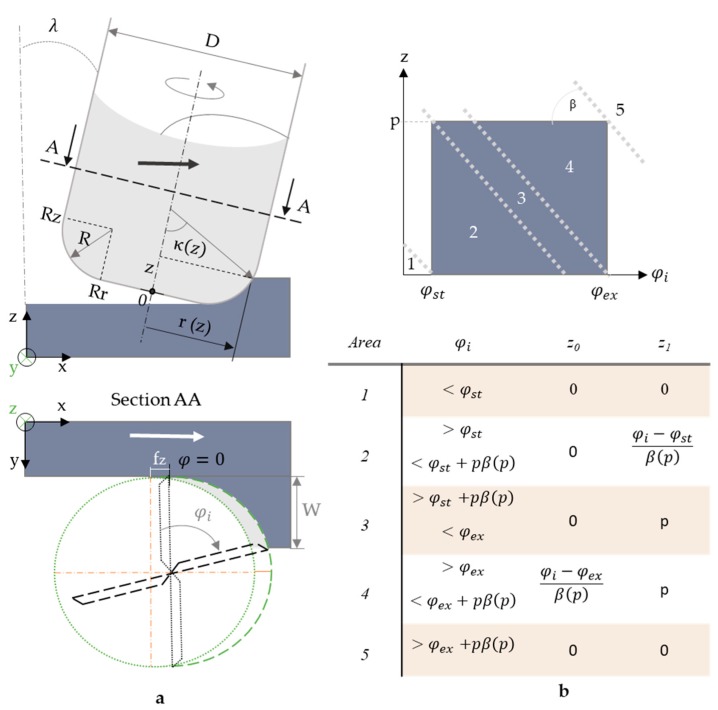
(**a**) Tool geometry; and (**b**) integration limits selection.

The expected forces can be calculated using a mechanistic model, which is adapted to the machining parameters, the tool, and the material as a function of the force coefficient. Tool geometry is very important, particularly to reduce vibration [1], and mechanistic models may be adapted to it. For this reason, Gradisek et al. [52] presented a generalization of the classical mechanistic model for most commercial tool geometries. Urbikain et al. [43,149] developed a specific model for a barrel tool shape, in which the positioning angle of the tool and runout—eccentricity of the tool due to its positioning on the head spindle—is also considered. Ma et al. [44] performed a similar work including the relative position of the tool for five-axis applications and its effect on dynamic stability. In this section, the mechanistic model is described using a standard bull end mill (Figure 4a) considering also the tool angle position (λ).

The force is obtained by integration of the differential edge elements contributing to the cut and summing the engaged teeth. The differential equation of the forces considering q={t,r,a} as the component tangential, radial or axial, respectively, can be defined as:

(1) $$\partial F_{q}\left(\varphi ,z\right)=K_{qe}\partial S+K_{qc}h\left(\varphi ,z\right)\partial b$$

where $K_{qe}$ comprises the force coefficients related to the friction phenomena and $K_{qc}$ the ones related to the cutting action. ∂b is the differential chip width and ∂S is the differential edge length. *h* is the chip thickness as a function of the rotated angle (φ) and the axial depth of cut (z).

(2) $$\partial b=\frac{\partial z}{\sin\kappa \left(z\right)}\;\partial S=\frac{\partial z}{\sqrt{1-{\left(\frac{R_{a}-z}{R}\right)}^{2}}}$$

The instant rotation angle is defined as:

(3) $$\varphi \left(\varphi_{i},z\right)=\varphi_{i}-\left(j-1\right)\frac{2\pi }{N}-\beta \left(z\right)$$

where *j* is the teeth number from 0 to N, considering N the total number of teeth and β(z) the helix angle as a function of the instant depth of cut, and can be defined as:

(4) $$\beta \left(z\right)=\left(1-\frac{R_{a}-z}{R}\right)\tan\beta_{0}$$

The instant chip thickness is defined as:

(5) $$h\left(\varphi ,z\right)=f_{z}\;\sin\varphi \left(\varphi_{i},z\right)\;\sin\kappa \left(z\right)\sin\lambda$$

The integration limits are determined by the starting and ending angle of engagement of the tool and the *z* minimum (*z*_0_) and maximum (*z*_1_) values applied on the instant angle (Figure 4b).

Forces in Cartesian coordinates can be calculated including the position angle of the tool.

(6) $$\frac{\partial F_{x,y,z}}{\partial z}=\left[\begin{array}{ccc} \sin\lambda & 0 & -\cos\lambda \\ 0 & 1 & 0\\ \cos\lambda & 0 & \sin\lambda \end{array}\right]\left[\begin{array}{ccc} -\cos\varphi & -\sin\varphi \sin\kappa & -\sin\varphi \cos\kappa \\ \sin\varphi & -\cos\varphi \sin\kappa & -\cos\varphi \cos\kappa \\ 0 & \cos\kappa & -\sin\kappa \end{array}\right]\left[\begin{array}{c} \partial F_{t}\\ \partial F_{r}\\ \partial F_{a} \end{array}\right]$$

Considering more than a single tooth, the total force applied is defined as:

(7) $$F_{x,y,z}\left(\varphi_{i}\right)=\overset{N}{\underset{j=0}{\sum }}\left[\int_{z_{1}}^{z_{2}}\partial F_{xyz,j}\left(\varphi_{i},z\right)\right]$$

The accuracy of those models depends on the fitting used for the calculation of the force coefficients ($K_{qc}$ and $K_{qe}$), and they are commonly predicted through experimental tests. Coefficient values are considered constants for different feed rate per tooth ($f_{z}$) while ∂b and ∂S depend on the tool geometry. The differential forces are studied on the Cartesian edges. Average forces for each test are used to calibrate the values of $K_{qe}$ and $K_{qc}$ for each condition of tool angle, material and spindle speed. Using at least two $f_{z}$, the six equations system is solved, obtaining the force coefficient. To improve the accuracy of the values, Liu et al. [105] used three spline interpolation. There are alternative methods to calculate $K_{qc}$ and $K_{qe}$ such as that of Du et al. [69], who used the Fourier form to determine the milling forces and then the force coefficient.

### 3.2. Dynamic Model

**Figure 5:**
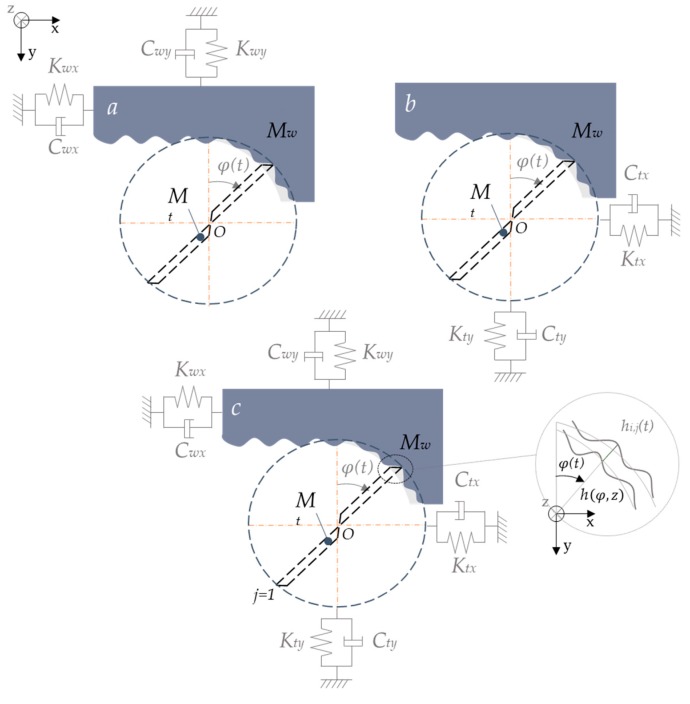
Flexibility of the system categorized as: (**a**) rigid cutter–flexible workpiece system; (**b**) rigid workpiece–flexible cutter system; and (**c**) double flexible system.

Once the cutting forces are calculated, stability of the system can be predicted based on the FRF and the cutting parameters values. To establish it, the degrees of freedom of the system are required. Tool and workpiece flexibility can be considered as independent, providing three different situations (Figure 5). The deflection of the part [10], the cutter system [64] or both [46] may affect the quality of the part in terms of final thickness and the roughness. For this reason, several researchers modeled the dynamic deformation of the system to predict the real quantity of material removed in each step and avoid reprocessing.

Most studies are based on the following assumptions:

- Temperature and other factors related to the machining process do not affect the behavior of the tool and the workpiece during the cutting operation.
- The only force considered is the cutting force, and deformation is only elastic.

Considering a mixed situation and a multiple contact model, the dynamic behavior of the system can be modeled by an equation of the form:

(8) $$M_{s}{\ddot{Q}}_{s}\left(t\right)+C_{s}{\dot{Q}}_{s}\left(t\right)+K_{s}Q_{s}\left(t\right)=F_{s}\left(t\right)\;\left(s=w,t\right)$$

where mass (***M****_s_*), damping (***C***_s_) and stiffness (***K***_s_) are matrices with dimension $3n_{s}\times 3n_{s}$ on the workpiece (*w*) and the tool (*t*). The vibration vector $Q_{s}\left(t\right)$ is defined by the modal displacement ($\Gamma_{s}\left(t\right)$) and the mass normalized mode (***U_s_***). $\zeta_{s}$ is the modal damping ratio matrix and $\omega_{s}$ is the diagonal FRF matrix, both matrices having the dimension of $m_{s}\times m_{s}$.

(9) $$\ddot{\Gamma_{s}}\left(t\right)+\left(2\zeta_{s}\omega_{s}\right)\dot{\Gamma_{s}}\left(t\right)+\omega_{s}^{2}\Gamma_{s}\left(t\right)=U_{s}^{T}F_{s}\left(t\right)$$

where

(10) $$\Gamma_{s}\left(t\right)=\left\{\begin{array}{c} \Gamma_{t}\left(t\right)\\ \Gamma_{w}\left(t\right) \end{array}\right\},\;\omega_{s}=\left[\begin{array}{cc} \omega_{t} & 0\\ 0 & \omega_{w} \end{array}\right],\;\zeta_{s}=\left[\begin{array}{cc} \zeta_{t} & 0\\ 0 & \zeta_{w} \end{array}\right],\;U_{s}=\left|U_{t}-U_{w}\right|,\;$$

(11) $$F_{s}=F_{t}=-F_{w}$$

*F_s_*(*t*) correspond to the cutting forces and is calculated following the force prediction section but, in this case, chip thickness ($h_{i,j}$) and axial immersion angle (κ) should consider the dynamic interaction:

(12) $$h_{i,j}\left(t\right)=\left[x_{i,j}\left(t\right)-x_{i,j}\left(t-T_{j-1}\right)\right]\sin\varphi \left(t\right)+\left[x_{i,j}\left(t\right)-x_{i,j}\left(t-T_{j-1}\right)\right]\cos\varphi \left(t\right)$$

(13) $$\kappa \left(t\right)=\cos^{-1}\left(\frac{V\cdot P}{{\left|P\right|}^{2}\left|V\right|\;}\right)$$

**Figure 6:**
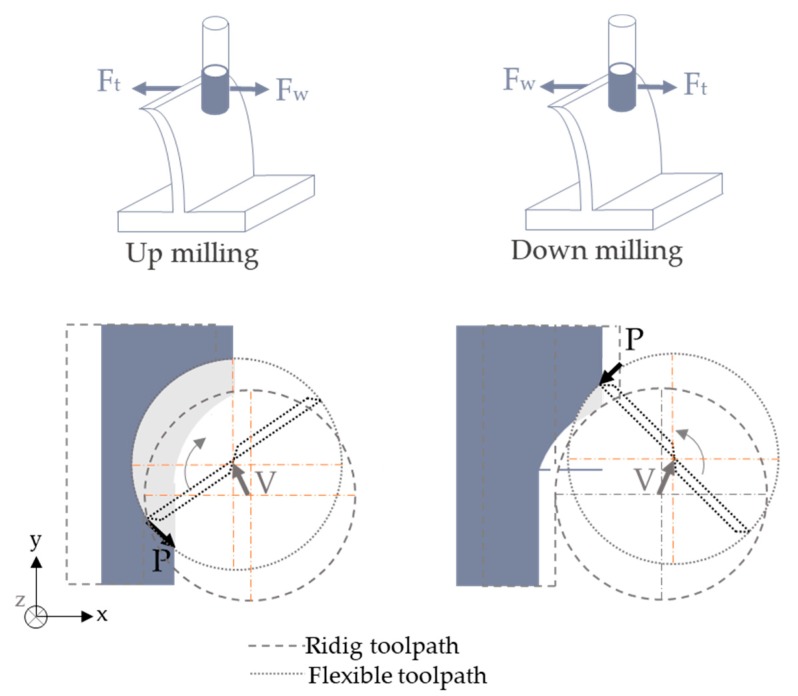
Deflection produced on the parts considering displacement of workpiece and tool.

*x* and *y* are the displacement between tool and workpiece during the effect of the *j* cutting flute at the time interval *t*. *T_j−1_* is the time interval between two following flutes. *V* is the tool axis vector and *P* is the relative position vector to the instant t, both of which depend on the instant relative position between the part and the tool (Figure 6).

(14) $$V=\left(V_{x},V_{y},V_{z}\right)\;P=\left(x_{0}-x_{i,j}\left(t\right),y_{0}-y_{i,j}\left(t\right),z_{0}-z_{i,j}\left(t\right)\right)$$

### 3.3. Deflection Model

Once the forces are stable and no chatter appears, deflection can still produce over cutting or under cutting. The normal force applied to the part can bend it depending on its stiffness (*k*), producing a displacement (*δ*) of the contact point between the workpiece and the tool.

(15) F=k δ

**Figure 7:**
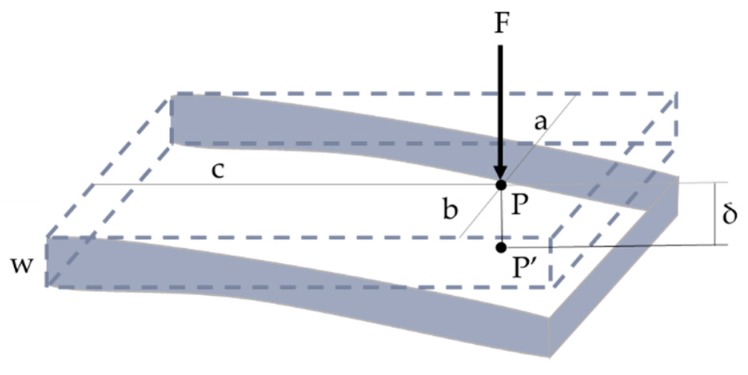
Scheme of the bending of thin-wall produced by the cutting forces.

Fixing the deflection, F can be related to the thickness of the part (Figure 7). The maximum value of the force is determined by the Young’s modulus (*E*), the Poisson ratio (*µ*), the position of the tool, and the total thickness (*w*). This thickness should be corrected considering the addendum to the real thickness depending on the location (Δw(u,v)), based on the tool referenced axis, and considering the residual thickness over the designed surface [66]. The final displacement is obtained based on the predicted forces. This model can be applied not only to cantilever plates but also to more complex geometries.

(16) $$F=\frac{E}{\left(1-\mu^{2}\right)}f\left(a,b,c\right){\left(w+\Delta w\left(u,v\right)\right)}^{3}$$

## 4. Computational Solutions

Computational analysis is used for the study of the final behavior of the part. It analyzes and predicts the vibration and deflection behavior during thin-wall milling. In both cases, initial forces are calculated using mechanistic models based on experimental data [64] or commercial software such as AdvanEdgeTM [70], VERICUT^®^ [148] or DEFORMTM [77]. They are used as inputs for the initial conditions of the workpiece. Then, self-developed or commercial software such as ANSYSTM [20,24,34,71] or ABAQUS [29,35,53,58,71,150] are used to obtain the FRF of the system, the dynamic behavior or its deflection.

### 4.1. Vibration Prediction

Different authors tried to establish new dynamic models based on computational experiments in order to predict chatter or forced vibrations during the machining of thin-wall parts. Most of them are based on the study of the FRF by analyzing Stability Lobes Diagrams (SLD) and instant chip engagement to choose the correct cutting parameters in order to improve surface quality of the part and reducing tool wear [21].

#### 4.1.1. Chatter

Chatter prediction starts by calculating the FRF of the workpiece and tool-spindle using impact hammer test [15,26,30,31,36,37,38]. One point of the tool and different locations of the workpiece are hit, and excitation responses are recorded by accelerometers. The weight of the accelerometers is subtracted from the total weight of the system. The data are treated and filtered to determine the FRF matrix and the modal damping ratio matrix. Damping ratios are usually considered constant during milling and FRF be studied under the most critical situations [3].

Those results lead to a general dynamic model that is dependent on the machining parameters. As is well known, machining parameters directly affect the efficiency of the process and, in this particular case, its stability. To ensure both of them, most researchers study the SLD of the system.

**Figure 8:**
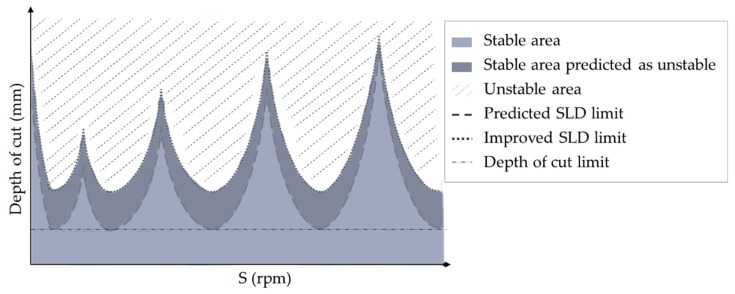
Schematic SLD presenting stable and unstable areas and the possible improvement of the SLD limit curve.

SLD are one of the most common tools used in thin-wall machining to select parameters in order to reduce chatter by just setting the correct machining parameters in terms of efficiency [39,40,41]. SLD usually represents the stability areas based on the axial depth of cut and the spindle speed (Figure 8). However, typical methods for calculating SLD determine more restrictive areas than they should [40,46]. This is the reason recent works focus on the improvement of its calculation.

Several authors [25,30,42,150] considered more than one parameter machining condition, generating 3D-SLD. Their studies mainly focus on sample parts to generalize monolithic part machining behavior. For instance, Olvera et al. [39], Germashev et al. [40] and Guo et al. [16] studied the runout and helix angle effect in a SLD, while Urbikain et al. [149] and Jing et al. [20] compared the effect of the tool position. Liu et al. [105] focused their research in the effect of the radial depth of cut and Qu et al. [25] analyzed the feed rate effect. Feng et al. [18] evaluated the effect of velocity-dependent mechanisms to obtain a closer SLD, obtaining better results than the ones using a plowing force coefficient. Finally, some studies are focused on the fixture effect, such as the flexible support [46] or the damping system [19,31,47].

However, few works were found where SLD approach is applied to mirror milling applications or similar test combinations [15,20]. For those cases, bull and ball end mills are used to analyze the effect of the angle or relative position of the tool in the SLD [15].

#### 4.1.2. Amplification

Resonance and amplification can be predicted based on the differential equations of the dynamic behavior of the system, which can be calculated through semi-analytical methods. These models have usually been developed in MATLAB [25,48,49] or C++ [45]. However, this solution is time consuming and has low accuracy. For this reason, one of the most recent approaches is to develop more efficient ways to solve the stability differential equations. Song et al. [50] used the Sherman–Morrison–Woodbury formula to calculate FRF considering the mass loss, whereas Li et al. [64] used a Runge–Kutta method for the same purpose. Feng et al. [18] used Taylor series to linearize the dynamic equations and Olvera et al. [39] solved the model using enhanced multistage homotopy perturbation (EMHP) and Chebyshev method in order to improve the accuracy.

**Figure 9:**
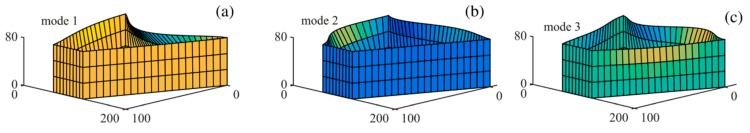
Average deflection obtained for (**a**) the first mode, (**b**) the second mode and (**c**) the third mode of a pocket structure [55].

Other authors use computational methods to predict vibrations. In this case, it is important to consider mass loss and tool wear because they can also modify the dynamics of the system and thus the stability of the machining [51]. This consideration implies a constant remeshing and reanalysis, involving a considerable computing time. Some authors tried to include the effect of the material loss. Meshreki et al. [151] proposed the use of 2D multispan plate (MSP). It improved the computational efficiency but it can only be applied to simple geometries. Budak et al. and Yang et al. [21,33] developed Structural Dynamic Modification (SDM) by updating the mass loss by the time domain. Tuysuz and Altintas [57] developed an iterated improved reduction system technique combined with a matrix perturbation technique to use the computational time only once. Yang et al. [29] used component mode synthesis (CMS) and space structural modification to develop a decomposition-condensation model that reduce computational time. Fei et al. [20] solved the dynamic model using a semi-discretization method. Ding et al. [19] established a dynamic model dividing the part and analyzing the FRF on both parts. Li et al. [64] improved roughness by developing a dynamic model for machining of integral impellers blades. Shuang et al. [59] used a coupled Eulerian–Lagrangian model to relate the chip formation to the cutting forces oscillation amplitudes, reducing the surface roughness produced by part deformation. The model used by Tian et al. [26] is presented as a theoretical base for suppressing resonance in the milling process. Ahmadi [55] compared a Finite Strip Model (FSM), FEM analysis and a semi-analytical model for the study of the dynamics of thin-wall machining (Figure 9). Lin et al. [56] studied the FRF of the machining system and related the waviness of the part with the force vibrations and not to the self-exited vibrations.

### 4.2. Dimensional Error Prediction

Computational methods for part deflection analysis usually involve simulations to estimate deflection of the part and reduce the dimensional tolerances of the process neglecting the dynamic response of the system [61].

Initially, the applied force is calculated based on mechanistic models and considering the instant chip formation. To improve the accuracy of the model, approaches such as the finite difference method are employed only for the contact interference between workpiece and tool [34] or Eulerian-Lagrangian methods to predict the chip formation and the final force applied [74].

Generally, the workpiece material is modeled using the Johnson–Cook law [74,78] and elastoplastic behavior should be considered [5]. Residual stress can be excluded from the material model since the induced residual stresses are more significant than those produced by previous forming steps such as rolling or forging [65]. However, their effect should be taken into account to predict the final part deflection.

**Figure 10:**
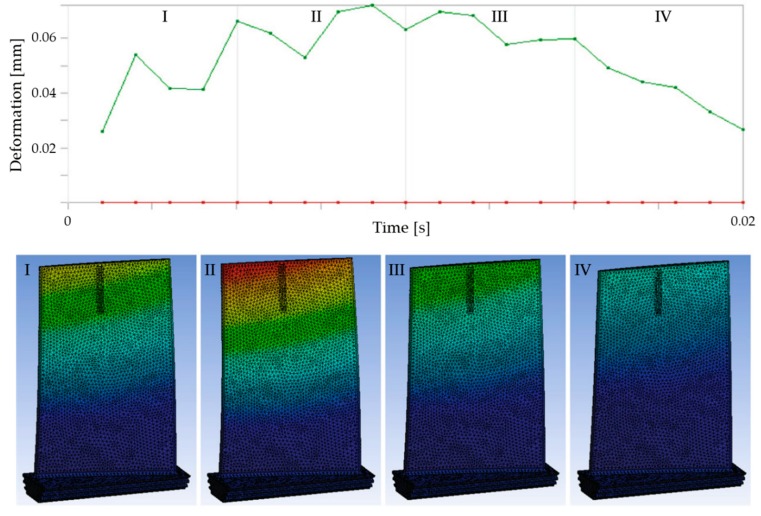
Deformation of a blade during one period rotation of the spindle [78].

The workpiece deflection is predicted through iteration and considering a quasi-static situation [61,70]. The analysis is performed following the toolpath and iteration must be performed for every new tool position due to the change of the workpiece–tool contact and the workpiece stiffness. In fact, the deformation produced by these changes can vary considerably just in one rotation of the spindle, as illustrated in Figure 10. Consequently, for each new position, the part should update the existing material, remeshing the workpiece and considerably increasing the computing time. Izamshah et al. [142] combined FEM and statistical models to reduce it in the simulation of the surface error. Ratchev et al. [62] addressed the solution by using a volume element-based surface generation approach to predict the deflection of the part. Similarly, Si-meng et al. [72] increased the solving speed by changing the simulation method. They considered the material loss using Boolean operations and hexahedral mapping algorithms, including tool and workpiece springback. Their models were validated with an error lower than 15%. Wang and Si [49] discarded the mesh subdivision or mesh adaptive technology because both considerably increased the computational burden, whereas the accuracy was not improved. Meanwhile, they simulated stiffness variation by removing the two elements adjacent to the cutter location, improving deflection accuracy.

Other authors focused on how the parameters affect the deflection of the part. Some of them studied the effect of the wall thickness [67,73], the depth of cut [66] or the feed rate [61,78] on the dimensional error. Others authors considered the tool position [49,70] and the fixture system [5]. Zhang et al. [28] went further by including the effect of the distance to the clamping system.

Another factor to take into account in the final dimensional error is the induced residual stress. It is usually evaluated for thin-walled parts through X-ray [79,80,81,82], neutron diffraction [83] or XDR [84] technologies. Residual stress can be affected by the cutting parameters, such as tool geometry [81,82], depth of cut [79], final quality, tool path [65], process temperature [84] and cutting forces [84]. In fact, the selection of the proper parameters with the aim of reducing the induced residual stress can lower the part deflection up to 45% [85]. For instance, Masoudi et al. [84] proved the effect of high-speed machining conditions on the reduction of the distortions produced by residual stresses, considering also that an increase of the depth of cut would increase the internal stress. Gao et al. [86] proposed a semi-analytical method to predict the deformation of thin-wall machining parts based on the effect of the residual stress present on the part. Jiang et al. [81,82] studied the uncut chip thickness effect on the induced residual stresses, relating also the strategy—up or down milling—and the change of tool diameter.

**Figure 11:**
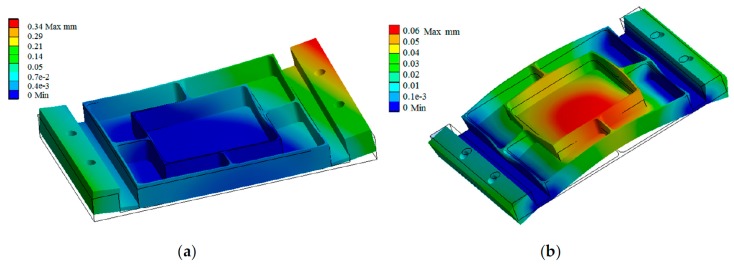
Deformation of a thin-wall part: (**a**) not considering the residual stress; and (**b**) following a quasi-symmetric machining reducing the residual stress [88].

Once the deflection is predicted, many methods apply the results to compensate the tool path or to modify the cutting parameters in order to reduce it [62]. Hao et al. [87] used this approach to correct blade deflections and to geometrically predict the roughness of the final part produced by the separation between the workpiece and the tool. Chen et al. [6] reduced the final error by applying a toolpath compensation strategy. Richter-Trummer et al. [12] presented a simulation method that predicted the distortion produced by residual stresses and allowed managing it, ensuring the dimensional quality of the machining parts. Similarly, Wu et al. [88] used quasi-symmetric machining to reduce the deformation produced by residual stresses. The machining results are considerably more accurate when the compensation is made at the last layer (Figure 11).

## 5. Industrial Approach

Apart from computational modeling, several solutions following an industrial approach have been developed such as parameter selection, adaptive control, or workholdings and fixture design.

### 5.1. Parameters Selection

Parameter selection can be established through experimental data-based models, commonly statistics or neural network based models, or through virtual twins studies, in which depth of cut and toolpath are modified to ensure the final thickness of the part.

#### 5.1.1. Database Models

Cutting parameters for thin-wall machining are also studied from an experimental and statistical point of view [13,89,90,91,92,93,94]. Researchers developed experimental models based on ANOVA results by analyzing how the cutting parameters and machining strategies affect the roughness and the dimensional error. Sonawane et al. [89] used ANOVA analysis to reduce the deflection of a cantilever sheet considering different inclination angles. Qu et al. [90] optimized the experimental models analysis using a neural network analysis type NSGA II. Izamshah et al. [142] obtained a generalized force model based on ANOVA analysis, training the dataset using FEM simulations. Oliveira et al. [91] found the milling strategy (down or up milling) as the most influencing parameter for dimensional error. They also remarked that f_z_ had an influence on surface roughness, but only when down milling strategies were used. Borojevic et al. [92] optimized the machining time based on the machining strategy and the cutting parameters. Bolar et al. [93] and Jiang et al. [7] detected three different areas of study for roughness when flank milling of thin-wall components was performed. The first area (initial engagement) and the last one (final disengagement) are more unstable than the center of the part. Both surface and residual stresses are increased due to the forced vibrations produced by the tool on those two areas. Yan et al. [66] implemented an experimental method that allow setting the maximum depth of cut as a function of the cutting force, thus its effect does not produce any displacement on the part.

All these models can be used as simplified models to implement on adaptive control, reducing time response and modifying the cutting parameters more quickly. The effect of the cutting parameters on residual stresses, cutting forces, deflection and surface roughness is summarized in Table 2.

#### 5.1.2. Virtual Twins

Another industrial approach is to develop virtual twins that will ensure the correct selection of parameters and machining strategies. Some CAM commercial software packages have optimization modules to integrate the dynamic error induced by the cutting forces as data but others only integrate the force and toolpath analysis on a FEM software. Rai et al. [5] considered elastoplastic deformation on a 3D virtual environment predicting the nonlinear behavior during machining. Jiang et al. [148] used the module VERICUT optimization to select the best parameters based on the part and tool model. This software uses neural networks to select the optimum set of parameters.

Under a set of conditions, constant thickness and cutting force, different tool paths are evaluated. Yan et al. [66] programed a depth of cut strategy simulating the physical behavior of a blade depending on the tool path generated on UG NX. The suggested variable depth of cut improves the machining error by up to 80% and save a third of the machining time. Rashev et al. [96] included artificial neural network to the CAM to improve the accuracy of the predicted deflection. Wan et al. [97] used deformation simulations to predict the optimum position of the support, evaluating the relative workpiece–part position.

Another application of virtual twins is to simulate the real position of the part. Especially mirror milling, due to their double curve, needs to use premeasuring techniques to redesign the tool-path considering the real position of the part [8,98,99]. Once it is calculated, software determines the tool path transplantation between the nominal surface and the actual one [98].

### 5.2. Adaptive Control

Adaptive control is a solution based on the on-line monitoring of the machining and an instant intervention on the process to ensure the final quality of the part. Signal data processing and monitoring systems are the base for automatic responses through parameter modification or active damping actuators. These options generally improve the vibration behavior of the system. Meanwhile, on-line measurement systems are used to reduce dimensional errors.

#### 5.2.1. Monitoring

The on-line detection of vibrations, combined with SLD data and dynamic or database models, can lead to adaptive control systems able to improve final quality of the part [106]. For that, a monitoring system able to distinguish the stability or instability of the process is required. Different authors have worked to implement filters and detection systems so parameter changes can be applied.

On the vibration field, Rubeo et al. [107] used the peak-to-peak force diagrams to detect instability. Germashev et al. [40] presented a simple FFT as a tool for prediction analysis and related the fluctuation of the tool with the surface quality. Tian et al. [26] proposed a matrix perturbation method as a time saving way to obtain the natural resonance frequency in thin-wall parts while they are machined. Tian et al. [45] used an eigenvalue sensitivity method to improve machining stability and the final surface finishing. Liu et al. [105] applied different filters to the cutting force in order to analyze the cutting coefficient behavior and its effect on the stability of the system. Liu et al. [108,109] used a Q-factor method to identify the change on the machining operation between the stable and chatter regions. The method was used for flank and mirror milling and quantified the level of chatter based on the force signal. Muhammad et al. [110] designed an active control system based on operational amplifier circuits where they can control the instant vibration, recording the acoustic signal with a microphone. Based on a dynamic model, the damping system changed the applied force so the chatter was reduced. Liu et al. [95] considered the deformation of the tool and the workpiece using an approach based on multisensor fusion and support vector machine (SVM) as a machine learning analysis. The recorded signal was analyzed using wavelet decomposition, and then SVM was applied to signal whether a change on the machining condition was needed. Ma et al. [111] developed a model to be implemented in adaptive control in which the feed rate was modified as a function of the real chip thickness. Feng et al. [32] established a different chatter model based on wavelet analysis of the cutting forces. They also studied its influence on the roughness of the part. Wan et al. [100] proposed a method for the chatter identification on thin-wall machining using a Hilberg–Huan transform and Gao et al. [101] used Complex Morlet Wavelet Transform (CMWT) to detect chatter in thin-wall machining.

Similarly, different techniques have been tested to prevent part deflection. On-line techniques have been developed based on signal treatment or on-line measurements. Wang et al. [102] used the lifting wavelet transform of the cutting forces to identify the bending of the part. Liu et al. [103] employed the cutting forces combined with a dynamic feature model that established the error compensation on real time to avoid the deformation. This solution considerably reduced the thickness error of the final part. Similarly, Han et al. [78] designed a parameter optimization federate control algorithm based on a previous simulation study of the deflection of the part that can be implemented as a control strategy schedule in an open modular architecture CNC system (OMACS). The control strategy schedule was based on the Brent–Dekker algorithm and it was successfully implemented as an adaptive control. Ma et al. [104] discovered the relationship between the induced residual stresses and the cutting power that can be used as a parameter in an on-line measuring method to avoid the deflections caused by residual stress.

#### 5.2.2. Measurements

As explained above, geometrical errors can be reduced by adjusting the depth of cut as a function of the real position of the part surface. However, the instant deformation of the part caused by the machining process is difficult to predict with pre-machining analysis, especially for complex components. Following this principle, some authors have proposed measuring the position of the workpiece on-line in order to ensure its final dimension and its surface quality.

Optical techniques were tested to define the cause of the part deflection [112]. Despite experiments related to the cutting conditions with the mechanical deformation, the acquired geometrical data were not used to reduce dimensional errors.

**Figure 12:**
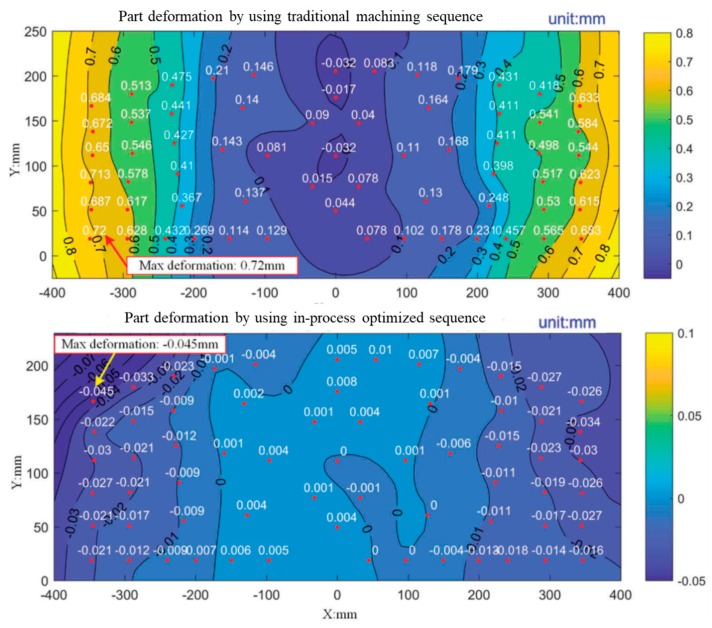
Part deformation obtained with a traditional machining sequence (upper) and with an adaptive machining system selecting an optimized sequence (lower) [106].

Touching displacement sensors have also been studied for on-line measurements. Wang et al. [113] adjusted the cutting depth according to the geometrical deviations of the thin-wall, which were measured before the finishing stage on the same milling machine where the rough machining was performed. The measurements allowed calculating the depth compensation value. Similarly, Hao et al. [106] reconstructed the real in-process surface using a displacement sensor. They developed an algorithm to adjust the toolpath and the machining sequence depending on the instant deformation of the part. Their results are shown in Figure 12.

Other authors have tested ultrasonic devices for this purpose. Huang et al. [114] automatically recalculated the new tool position combining ultrasonic on-line measures and touching measures of a tank bottom. Rubio et al. [115] designed a flexible clamping system with an ultrasonic premeasure device that automatically adjusted the depth of cut to ensure the final thickness of the skin.

In the same way, Wang et al. [116] used a laser displacement sensor included on the supporting head to measure the in-process displacements of the workpiece. They implemented a forecasting compensatory control system to predict the skin deflection and to adaptively control the width of cut improving the quality of the final part.

### 5.3. Fixtures, Workholdings and Stiffening Devices

A different strategy to prevent instabilities during low rigidity part machining is to address this problem from the fixing perspective. This strategy could be more efficient than selecting free chatter cutting parameters for complex parts, as FRF is difficult to obtain and it is continuously changing during machining operation.

#### 5.3.1. Fixtures and Workholdings

One of the most common fixture systems for thin-wall parts are vacuum fixtures [83,116,117,118,119,120]. Vacuum fixture applications are based on two main two options: customized vacuum systems or flexible vacuum cups. Their use can reduce vibration and deflection [27]. On the one hand, customized vacuum systems use special vacuum adsorption equipment for each part to be machined [120]. This option is expensive and limited to the number of equal parts produced. Moreover, the vacuum table can produce a tensile stress on the part, affecting the part deformation [12,83]. On the other hand, adaptable vacuum cups or beds increase the flexibility of the clamping fixture. This solution is based on flexible pins combined with vacuum caps or heads that adapt the position to the curves of the part [121]. Some researchers have used the virtual twin concept to select the positions of the additional supports needed for these parts [122,123,124,125,126]. They simulated the system obtaining a minimum part deformation. However, for large skin panels, the support provided by the cups is not enough to reduce deflection or could need a complex optimization of the cup position to reduce it [76,117]. For this reason, Rubio et al. [127] developed a flexible vacuum bed insuring the contact between the part and the workholding, reducing both vibration and deflection.

**Figure 13:**
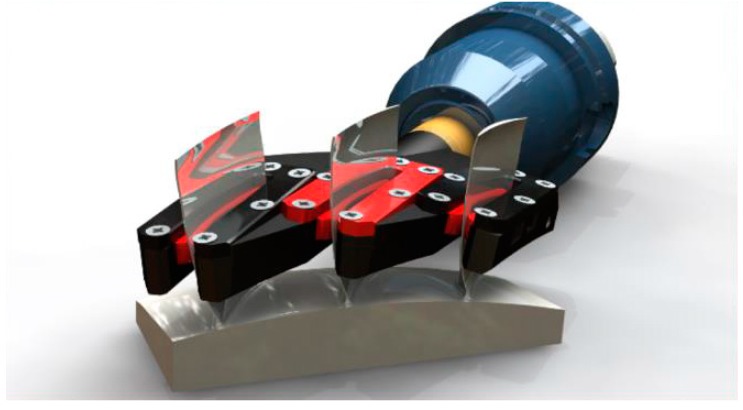
Innoclamp^®^ holding system [128].

Impellers, blades and blisks are usually clamped using hydraulic chunks or special jaws that try to reduce the clamping pressure, reducing the possible in-process deformation. These systems can avoid vibrations and deflection for the initial roughing steps but machining performance can be improved using additional workholding. To increase the machining parameters and the operation efficiency, flexible workholdings have been designed for the machining of thin-wall parts. These devices apply a support on the predicted optimal position based on FEM studies [24,124]. Figure 13 shows a commercial example of the company INNOCLAMP^®^ [128]. The workholding is specially designed to compensate the cutting energy all over the part. The position is defined using simulations, and the supports are applied at the most flexible positions. The system usually has embedded sensors, thus it is possible to change the behavior of the workholding, depending on the operation and to register historical data to feed new databases.

Alternatively, moving fixtures are used to maintain stability during low rigidity part machining. Most of the solutions are based on the mirror milling concept, a supporting element that moves synchronously to the tool. For instance, Fei et al. [48] designed a supporting element that moves collinear to the tool-path, acting as support of the cutting force. They compared the results with and without the fixing element, verifying the suppression of the deformation during the machining and the decrease of vibration amplitude, improving consequently the final roughness. Similarly, Liu et al. [129] proposed the use of an air jet to reduce deflection on the part. The jet was synchronized with the machined head following the mirror milling criteria. It impacted on one side of the wall, acting as a support of the cutting forces. Its effect was evaluated through vibration, cutting forces, thickness and final roughness data. Cutting experiments proved that the air jet assistance reduced the vibration of the workpiece up to 47% and both, thickness error and machined surface quality, were improved. However, both systems [48,129] are used for sample parts and the flexibility of it application is questionable, especially for complex geometries.

Mahmud et al. [75] tested a more sophisticated moving fixture to hold a fuselage panel, while skin panel pockets were milled. A magnetic workholding, made up of two sets of magnets, held the fuselage panel. The master set of magnets was placed on the mill tool side, following the tool trajectory provided by the milling machine. The slave set was located on the fuselage panel back and it followed the master module, compensating the cutting forces by the magnetic attraction force. It was experimentally verified that magnetic forces supported the milling thrust force and they overcame the frictional force on the slave unit.

Similar fixing systems were designed by machine tool makers such as Dufieux [130] and M. Torres [131]. They developed a mirror machine center provided with a double-head mechanism. The cutting tool installed on one head is used to remove materials from one side of the skin. Meanwhile, a twin head moves simultaneously providing an auxiliary support while the skin thickness is measured on-line to control the tool position and the depth of cut along the tool trajectory. Even though these solutions have already been implemented at industrial level for fuselage panels milling, they are still limited due to their high investment costs and need to use premeasuring techniques to redesign the tool-path considering the real position of the part.

#### 5.3.2. Active Damping Actuators

Active dampers have the ability to accommodate variable conditions where they are more difficult to implement. It is a specific part of the adaptive control in which the main objective is to avoid vibrations. In this case, the adaptive control makes the decision based on the monitoring system and actuates through piezo-actuator sensors or eddy current damping (EDC). Although active damping is more difficult to implement in real industrial environments, using this approach, a sevenfold improvement in the limiting depth of cut can be obtained. For example, Zhang and Sims [132] reported workpiece chatter avoidance in milling using piezoelectric active damping mounted directly on the workpiece. The research was done on simple geometry parts such as a cantilever plate. Diez et al. [133] used this technology to compensate the deformations of the part, improving final dimensional error. Rashid and Nicolescu [134] proposed an active control of workpiece vibrations in milling through piezo-actuators embedded in workholding systems. Although the tested part had a simple geometry (rectangular blocks) and it was dynamically stiff, the vibration active control improved the dynamics of the workholding system by cancelling the vibration signal generated by the cutting process, succeeding in improving surface quality and tool life.

Yang et al. [135] designed an active and lightweight device based on EDC to attenuate the vibrations produced during the machining of a thin-walled aluminum frame. They attached an ECD to the workpiece, which vibrated synchronously to it. Based on the electromagnetic induction, when a relative motion against the magnet appears, the magnetic flux through a conductor changes and a repulsive force is generated, attenuating the vibration. ECD demonstrated the capacity of keeping the process stable under different cutting parameters (f_z_, S, and a_p_), achieving a reduction of the machining vibrations of up to 84%.

#### 5.3.3. Stiffening Devices

Stiffening devices, opposite to active damping, add an extra device to the flexible component such as mass compensation system [136], magnetorheological fluids [47,137] or low melting alloys [138]. Their main advantages are the simplicity of their design and their easy implementation. However, they do not consider the stiffness change.

Trying to increase the rigidity of the part, Diaz et al. [137] investigated the use magnetorheological fluids (MRF) to prevent instabilities during thin-floor parts machining. These types of fluids have the ability to change from liquid to a semi-solid state due to the action of a magnetic field. Instead of applying variable cutting conditions to avoid chatter, they proposed the use of a shock absorber based on MRF. The way to assembly the shock absorber to the workpiece, the amount of fluid and how much voltage needed to be applied are issues of vital importance to make the shock absorber work correctly. Experiments proved it was possible to reach optimum machining speeds in the absence of instabilities during the machining of a thin-floor part. Wang et al. [138] investigated the use of a low-melting point alloy (LMPA) as a phase change material to configure flexible fixtures. This research was focused on complex thin-walled parts, which are difficult to clamp due to the low thickness of the walls. The LMPA was heated up to 70 °C—its melting point—and casted in the gap between the part and a rigid fixture to form a rigid body with fixture among the LMPA, the fixture and the part. The LMPA increased the rigidity of the component during machining. It also reduced the deformation and the vibrations caused by the milling forces, significantly improving the machining accuracy. Furthermore, once the LMPA was melted again, almost no impact was observable on the workpiece.

Other authors have focused their studies on mass compensation systems. Kolluru et al. [139] added a viscoelastic passive dampers to minimize machining vibrations in a ring-type workpiece. A viscoelastic tape (3M^®^ ISD112) was used to place the tuned damper blocks. The viscoelastic tape thickness, its weight and the position of the tuned mass were optimized using FEM. The dynamic response of the workpiece with and without dampers was simulated and the predicted responses were validated by impact hammer tests. The efficacy of dampers blocks was evaluated by machining undamped casing and damped casing and their use provided a significant reduction in vibration in terms of root mean square error. The author affirmed this solution can be rapidly adapted to other workpiece geometries using the FEM model they developed.

A similar approach was reported by Woody et al. [140], who addressed the potential of energy absorbing urethane foams as a passive damping to improve the dynamic behavior of an open-back. Different damping configurations were simulated and tested by FEM and experimental analysis. FRF measurements proved that the energy absorbing foam fabricated from urethane increased the damping performance by sixty times, adding less than 6% of mass compared to the overall structure. Both studies [139,140] agreed that the position of the material was important to define the clamping system, being the highest weight the most relevant factor.

Nevertheless, once a specific step of the machining process is reached, it is possible to detect a decrease on the efficiency of the damper compensating the FRF of the system. For this reason, Yang et al. [136] proposed a passive damper with tunable stiffness to suppress the wide range of vibration frequencies covered on the thin-wall machining. The ratio between the FRF of the passive damper and the workpiece varies with the material removal, especially for the thin-walled part. For this reason, they located a damper inside the workpiece and changed its orientation, modifying the workpiece stiffness. The amplitude of the workpiece FRF at the target mode was reduced by 40%, decreasing the machining vibrations and the surface roughness.

## 6. Conclusions

This work presents the state of the art of thin-wall machining. This machining usually presents dynamic and static problems that should be controlled to ensure the final quality of the part. Analytic models allow understanding the behavior of the system, defining where vibration or deflection appear. They are also the base of the computational solutions where the most significant advances have been focused on including mass loss and stiffness variation, reducing computational time and increasing prediction accuracy.

Industrial approach have been focused on the following:

- Virtual twins development integrates CAM and simulation to predict the machining behavior, the future real position of the surface to cut, and improve the machining efficiency by selecting the proper cutting parameter, toolpath, or prediction [152].
- Adaptive control, which is used in production to improve the part quality and to feed the database models, detects the process instabilities or deformation through signal analysis and changing on-line the machining parameters.
- Fixture systems design and new approaches try to include adaptive control on the workholding or the stiffening devices to increase the product efficiency, allowing to use more aggressive cutting parameters.

Overall, these studies highlight the need for more accurate simulations and control of the machining process [153]. On the one hand, there remain several aspects of computational modeling about which is little known, particularly computational time reduction is needed to consider the simulation of complex final geometries.

On the other hand, a roughness control method should be designed to detect on-line vibration and deflection with a shorter time response. For example, real-time networks could be implemented using an Internet of Things approach to collect and treat data. The improvement of interfaces and connectivity could lead to avoiding reprocessing steps. Intelligent machine control could also be achieved through machine learning or metaheuristic algorithm analysis of big data. New processing schemes can be investigated for machining time and part deformation reduction, such as simultaneous machining for monolithic parts. Considering all of this evidence, it seems that industry 4.0 outlines are not completely integrated and their integration could make thin-wall machining more profitable.

## Figures and Tables

**Table 1:** Thin-wall machining solutions. Model and industrial approaches.

**Models**		
Thin-wall dynamic problems	Chatter and self-exciting aspects	[14,15,16,17,18,19,20,21,22,23,24,25,26,27,28,29,30,31,32,33,34,35,36,37,38,39,40,41,42]
	Resonance and amplification	[33,41,43,44,45,46,47,48,49,50,51,52,53,54,55,56,57,58,59,60]
Thin-wall deformation	Quasi-static models	[36,49,61,62,63,64,65,66,67,68,69,70]
	FEM modeling	[51,61,62,65,71,72,73,74,75,76,77,78]
	Residual Stresses	[79,80,81,82,83,84,85,86,87,88]
**Industrial Approach**		
Parameter selection	Statistic and machine learning models	[62,89,90,91,92,93,94,95]
	Virtual Twins	[66,78,96,97,98,99]
Active solutions	Monitoring	[32,41,95,100,101,102,103,104,105,106,107,108,109,110,111]
	Measurements	[106,112,113,114,115,116]
Fixture and clamping	Fixtures	[83,116,117,118,119,120,121,122,123,124,125,126]
	Workholding	[19,75,127,128,129,130,131]
	Active damping actuators	[132,133,134,135]
	Stiffening devices	[136,137,138,139,140]

**Table 2:** Cutting parameters effect on residual stress, forces, deflection and roughness. S, Spindle Speed; f, feed rate; Ap, depth of cut; NP, Nº of paths; MRR, Material Removal Rate; RS, Residual Stress; F, Forces; Def, Deflection; Rg, Surface Roughness.

	RS	F	Def	Rg
S	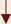	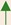	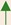	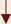
f	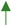	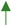	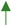 / 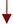 _1_	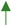
Ap	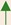	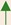	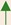	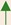
NP	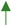		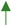	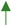
MRR		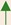		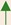

^1^ Down milling strategies increase the deflection of the part while up milling decrease it.
